# Cerebral vascular anomalies in a large Italian family with late-onset glycogenosis II

**DOI:** 10.1186/1471-2474-14-S2-P10

**Published:** 2013-05-29

**Authors:** F Cipullo, S Sampaolo, O Farina, M Simonetti, M Cirillo, G Di Iorio

**Affiliations:** 1 Dipartimento di Scienze Mediche, Chirurgiche, Neurologiche, Metaboliche e dell’Invecchiamento - Seconda Università di Napoli, Naples, Italy

## Introduction

Basilar artery dolichoectasia, ectasia of internal carotids and intracranial aneurysm have been described in late-onset glycogenosis II (GSD II). Incidence of these anomalies and their relationship to the genotype are unknown. Also, because studying cerebral vessels is not recommended for diagnosis of late-onset GSDII, the frequency of these anomalies may be underestimated. Therefore, we investigated the occurrence of vascular anomalies in eight siblings (4M, 4F) of an Italian family affected by late-onset GSDII due to a compound mutation (c.118C>T in exon 2 and c.2647-7G>A in intron 18) of the acid α-glucosidase (GAA) gene (17q21-23).

## Results

The clinical picture in these siblings is typical of late-onset GSDII: Patients never complained of symptoms of cerebro-vascular disease and none had cardiac involvement. Blood pressure and blood glucose were normal. Brain Magnetic Resonance Angiography (MRA; Angio-RM TOF 3D axial Siemens Synphony 1.5 T) was performed in 7 (4M, 3F) out of 8 patients (one female carried a paramagnetic prosthesis), and in 10 controls. Vessel total square diameter was measured at the apex and middle third of the basilar artery and at the middle portion of both right and left internal carotid artery. Blind measurements were carried out by three investigators. Basilar artery dolichoectasia (0.06% in the general population) was found in 3M and 2F, and a slight dilatation of internal carotids compared to controls was found in all patients.

## Conclusion

These data confirm that anomalies of main cerebral vessels are relatively frequent in late-onset GSDII patients. The role of genotype in determining these anomalies is uncertain because studies on informative families are rare. Particularly intriguing is the relation of these vascular anomalies with the risk of stroke in these patients. Even though all have ventilatory insufficiency with reduced blood oxygen saturation, nocturnal apnea, and impaired glycogen metabolism, a minority suffer TIA or stroke. Therefore, an imaging study of the cerebral circulation is recommended in all patients with late-onset GSDII. In those with anomalies, further research is needed to evaluate cerebral metabolism and to search for specific vascular risk factors.

